# Mechanism and Modeling of Moisture-Dependent Dielectric Properties of Cement-Based Composites for Enhanced Ground Penetrating Radar Applications

**DOI:** 10.3390/ma19081528

**Published:** 2026-04-10

**Authors:** Tao Wang, Bei Zhang, Yanlong Gao, Xiao Wang, Di Wang

**Affiliations:** 1 School of Water Conservancy and Transportation, Zhengzhou University, Zhengzhou 450001, China; wt1020@163.com (T.W.); 13155486232@163.com (Y.G.); 2 School of Materials Science, North China University of Water Resources and Electric Power, Zhengzhou 451460, China; wxzd0098@163.com; 3 Zhengzhou Anyuan Engineering Technology Co., Ltd., Zhengzhou 450048, China; wangdijiaotong@163.com

**Keywords:** cement-based composites, dielectric model, relative humidity, permittivity, GPR, non-destructive testing

## Abstract

The dielectric properties of cement-based composites (CBC) are highly sensitive to environmental humidity, which seriously restricts the quantitative interpretation accuracy of ground-penetrating radar (GPR) in the non-destructive testing of cement concrete pavement. In view of the lack of targeted prediction models due to the unclear mechanism of humidity influence in existing research, the core innovations of this study are: (1) the synergistic mechanism of water vapor dipole polarization and adsorbed water multi-layer polarization is clarified, revealing the intrinsic reason for the accelerated growth of permittivity in the high humidity range; (2) the constructed four-component dielectric model of “cement mortar–aggregate–water vapor–adsorbed water” achieves high-precision prediction within the range of 50~100% RH (R^2^ > 0.94, relative error < 5%), and shows good predictive ability within the test scope of this study; (3) a GPR humidity correction protocol based on the model is proposed, which can effectively improve the accuracy of nondestructive testing of cement concrete structures. In this study, CBC samples with water–cement ratios of 0.4~0.6 were prepared using P.O 32.5/P.O 42.5 cement and limestone aggregate. Under the conditions of 20 ± 0.5 °C, relative humidity (RH) of 50~100%, and 2 GHz (common GPR frequency), the permittivity was measured using an Agilent P5001A network analyzer to verify the model. The results show that the permittivity increases monotonically with humidity, and the growth rate in the high humidity range (70~100%) is 2.2 times that of the low humidity range (50~70%); The higher the water–cement ratio, the shorter the age, and the lower the cement strength grade, the stronger the humidity sensitivity of CBC dielectric properties. This model provides a reliable humidity correction tool for GPR detection, and significantly improves the accuracy of nondestructive evaluation of cement concrete structures.

## 1. Introduction

As an important part of modern transportation infrastructure, cement concrete pavement is widely used in key engineering fields such as highway and airport runway due to its high strength, durability, and low maintenance cost. However, with the increasing traffic load and the complexity of environmental conditions, the performance degradation of cement concrete pavement has become increasingly prominent. In particular, the long-term impact of moisture infiltration and humidity change on pavement structure can not be ignored. As an efficient, fast and non-destructive testing technology, GPR has been widely used in pavement quality assessment. Its core principle is to infer the dielectric constant of the pavement by analyzing the propagation characteristics of electromagnetic waves in the medium, and then to evaluate the key performance indicators such as the thickness, water content, and porosity of the structural layer. However, the dielectric properties of cement concrete are affected by many environmental factors, in which humidity is an important variable. Humidity not only directly affects the moisture distribution and content in cement concrete, but also significantly affects its dielectric properties by changing the microstructure of the material. Therefore, in-depth study of the influence of relative humidity on the dielectric properties of cement concrete is of great significance to improve the GPR detection accuracy and optimize the pavement maintenance strategy.

Permittivity is the most important parameter of the dielectric properties of road materials, and also one of the most important material properties reflected by GPR data [1]. The formula describing the relationship between the permittivity of composites and its component permittivity or volume ratio is called the dielectric model [2]. Based on this model, the dielectric properties and main influencing factors of composites can be understood in more detail, so as to support the relevant theoretical analysis.

Early classical dielectric models are mainly divided into volume mixing models and effective medium theory, but they are mostly applicable to two-phase composites, without considering the multi-phase characteristics of cement-based composites and the dynamic influence of humidity, and cannot be directly used for humidity correction in GPR detection [3,4,5,6,7,8,9,10,11].

Numerous studies have focused on concrete mix proportion, related mechanical properties, durability, constitutive relationship, and dielectric model [12,13]. Amara Loulizi studied the influence of cement concrete composition, salt, freeze–thaw cycle, alkali aggregate reaction, and other factors on the dielectric properties of cement concrete at the end of the 20th century, and proved that the permittivity of cement concrete mainly depends on the mixture composition and water cement ratio [14]. Al-Mattarneh successfully predicted the chloride content in concrete [12]. He et al. [15] found that chloride ion content also affects the dielectric properties of cement concrete. Water has a permanent dipole, and the permittivity at 25 °C is about 81, while the permittivity of cement concrete is about 3–8 [16]. Therefore, the internal water of concrete has a significant effect on the permittivity. Experiments are being used to establish the relationship between water content and dielectric properties. Laurens et al. [17] used 1.5 GHz ground penetrating radar to measure the electromagnetic wave amplitude change (hydration and drying) of cement concrete in the process of free water consumption, and found that the propagation of electromagnetic waves on the sample surface is closely related to the moisture change of cement concrete, which can be used to evaluate the volumetric moisture content [18]. The same method is used to explore the relationship between permittivity and water content [3]. Synthetic Aperture Radar (SAR) uses the principle of synthetic aperture to achieve high-resolution microwave imaging, which is used to monitor the change of concrete moisture content. The measured image can not only locate the distribution of water, but can also calculate the total content. The analysis shows that they have a linear relationship [19].

## 2. Influence Mechanism of Humidity on Dielectric Properties of Cement-Based Composites

According to relevant data, cement concrete, as a typical multiphase porous composite, has a porous medium structure inside, and the pore system (such as capillary pores, gel pores, etc.) provides a channel for the storage and migration of water. When the environmental humidity changes, water exchanges with the outside through the pores, and exists in the form of gaseous water molecules, liquid water, adsorbed water, etc., which leads to dynamic fluctuations in the internal humidity of concrete, and then affects its dielectric properties.

There is a continuous water transmission process in the cement concrete. Water exists in various forms such as gas and liquid in the concrete pore structure, and continuously exchanges with the external environment. This dynamic change of moisture leads to the dynamic fluctuation of moisture in cement concrete during service. The change of moisture will significantly affect its dielectric properties, and will then affect the accuracy of non-destructive testing technology based on the dielectric principle.

The pore system in cement concrete provides space for water storage and migration. When environmental humidity changes, water will exchange with the outside through pores. In this process, the existence, form, and distribution of water molecules will affect the dielectric properties of concrete. Water molecules are polar molecules and will be polarized under the action of an electromagnetic field. The influence mechanism of humidity on the dielectric properties of cement-based composites is the synergistic effect of water vapor dipole polarization and adsorbed water multi-layer polarization, which can be divided into three stages according to humidity ranges:

(a) Low humidity range (50~70% RH): Moisture in pores mainly exists in the form of water vapor and monolayer adsorbed water. Water vapor molecules are dispersed and move freely in pores. Although a single gaseous water molecule has polarity, its impact on the overall dielectric properties of concrete is relatively small due to dispersed distribution and weak interaction. Monolayer adsorbed water binds to hydroxyl groups on the surface of hydration products through hydrogen bonds, and its polarization intensity is weak. Therefore, the permittivity grows slowly in this range.
(b) Medium-high humidity range (70~90% RH): With the increase of humidity, the water vapor concentration in pores increases, and multilayer adsorbed water is gradually formed on the pore surface. Water molecules in the adsorbed water layer interact with each other through hydrogen bonds to form a continuous water film. Under the action of an electromagnetic field, the polarization process of the adsorbed water film is more intense, and the superposition of multi-layer polarization significantly enhances the overall polarization intensity of the material, leading to an accelerated growth rate of the permittivity.
(c) High humidity range (90~100% RH): When the relative humidity approaches saturation, capillary condensation occurs in large pores, forming liquid water microregions. The permittivity of liquid water (about 80.2 at 20 °C) is much higher than that of other components. The existence of liquid water not only increases the content of polar substances in concrete but also forms continuous polarization channels, which further enhances the polarization intensity and leads to a rapid growth of the permittivity [20].

In addition, the chemical composition and microstructure of cement concrete will also interact with water to further affect the dielectric properties. Cement hydration products have certain water absorption, and water molecules will be adsorbed on the surface of hydration products or enter their interlayer structure, which will change the surface properties and internal charge distribution of hydration products, thus affecting the dielectric properties. Moreover, under different humidity conditions, the behavior of ion migration and diffusion in concrete will also change, and the movement of ions under the action of an electric field will produce an additional dielectric effect, which will contribute to the overall dielectric properties.

Due to the influence of moisture movement, cement concrete can be considered to be composed of cement mortar, aggregate, and moisture (air containing water molecules). Due to the limitations of the non-destructive testing environment and equipment conditions, the influence of liquid water on the dielectric properties of cement concrete is not considered in this study, and the influence of water vapor concentration in cement concrete on its dielectric properties will be studied. In this study, the liquid water microregion formed by capillary condensation is considered to be in the range of 90~100% RH, but unlike free liquid water, the liquid water microregion is formed by adhering to the pore wall and is closely connected with the adsorbed water film, and its dielectric contribution has been indirectly incorporated into the model through the correction of adsorbed water phase parameters. What is not considered in this study is the free flowing liquid water in the macropores, as there is no such free liquid water in the cement concrete pavement in the non-ponding state in engineering practice. Therefore, the assumptions of the model are highly matched with the actual engineering detection scene.

**Figure 1:**
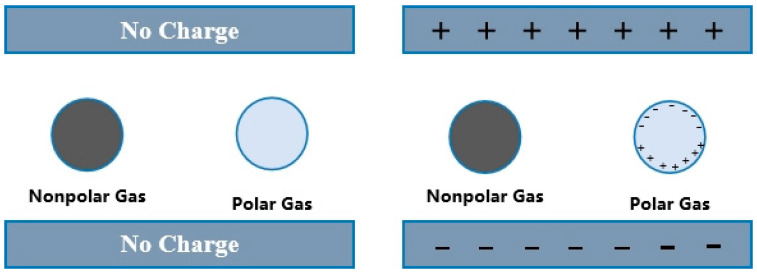
Schematic diagram of the influence of electromagnetic fields on polar and non-polar gas molecules.

Air is a mixture of polar and nonpolar gases. Under the action of electromagnetic field, polar gas will have molecular polarization, while non-polar gas will not [21]. The schematic diagram of polar and non-polar gas molecules affected by an electromagnetic field is shown in Figure 1.

## 3. Construction of Dielectric Model Considering Humidity

### 3.1. Relationship Between Polarization Intensity and Permittivity

According to electromagnetic theory, the polarization strength P is defined as the total electric dipole moment per unit volume, expressed as [22]:

(1) P=Np

In the formula, *N* is the number of dipoles per unit volume (m^−3^), and *p* is the electric dipole moment of a single dipole (C·m)

The relationship between the electric dipole moment p and the local electric field *E_loc_* is:

(2) $$p=\alpha \varepsilon_{0}E_{loc}$$

In the formula, *α* is the molecular polarizability (m^3^).

Combining Equations (1) and (2), we obtain:

(3) $$P=N\alpha \varepsilon_{0}E_{loc}$$

For a homogeneous medium, the relationship between the local electric field *E_loc_* and the macroscopic electric field *E* is:

(4) $$E_{loc}=E+\frac{P}{3\varepsilon_{0}}$$

By substituting Equation (3) into Equation (4) and combining $P=\varepsilon_{0}\left(\varepsilon_{r}-1\right)E$, we can summarize.

(5) $$\varepsilon_{r}-1=3N\alpha \left(3+N\alpha \right)$$

Equation (5) is the Clausius–Mossotti equation, which is the core relationship between permittivity and molecular polarizability. Dimensional check: the dimension of *N_α_* is $m^{-3}\cdot m^{3}=1$ (dimensionless), and both sides of the equation are dimensionless, which meets the requirement of dimensional consistency.

### 3.2. Four Phase Mixed Dielectric Model

The Brown mixture model (a variant of the logarithmic mixture law) is applicable for predicting the permittivity of multiphase composite materials, and has been widely validated in cement-based materials [23]. Its expression is:

(6) $$\ln\varepsilon_{r}=\ln v_{1}\varepsilon_{1}+\ln v_{2}\varepsilon_{2}+\ln v_{3}\varepsilon_{3}$$

Among these, *v*_1_, *v*_2_, and *v*_3_ are the volume fractions of cement mortar, aggregate, and water vapor, respectively ($v_{1}+v_{2}+v_{3}=1$); *ε*_1_, *ε*_2_, and *ε*_3_ are the relative permittivity of each phase, respectively; *K* is the model fitting parameter.

Combining Equation (3), the contribution of water vapor to the polarization intensity ***P***_3_ can be calculated as:

(7) $$P_{3}=N_{3}\alpha_{3}\varepsilon_{0}E_{loc}$$

Among these, *N_H_* is the number of dipoles introduced per unit volume of absolute humidity, and *α_H_* is the polarizability introduced per unit of absolute humidity. Assuming that the local electric field does not vary with absolute humidity under test conditions, *N_H_α_H_* can be expressed as a constant *K*:

(8) $$P_{H}=K\varepsilon_{0}E_{loc}$$

*n* the basis of Equation (6), and an adsorption aqueous phase is added to construct a four phase mixed dielectric model for cement-based materials:

(9) $$ln\varepsilon_{r}=v_{1}\ln\varepsilon_{1}+v_{2}\ln\varepsilon_{2}+v_{3}\ln\varepsilon_{3}+v_{4}\ln\varepsilon_{4}$$

The newly added parameters are defined as follows:

*v*_4_ (adsorbed water volume fraction): calculated by combining pore structure data (total porosity, specific surface area) with adsorption isotherm results v4=ma·ρwater−1Vs, where *m_a_* is the adsorbed water mass per unit sample mass and Vs is the sample volume.

*ε*_4_ (permittivity of adsorbed water): related to the thickness of the adsorbed water film (*d*) and the permittivity of the liquid water ($\varepsilon_{li}q_{ui}=80.2$), expressed as: $\varepsilon_{4}=\varepsilon_{li}q_{ui}\cdot {\left(d/d_{0}\right)}^{\lambda }$.

Among which:

*d*_0_ = 0.3 nm (Single layer adsorbed water thickness, fixed value based on molecular dynamics simulation [24]);

*λ* = 075 (Polarization attenuation coefficient of adsorption layer, determined by fitting experimental data, reflecting the weakening effect of polarization intensity with increasing film thickness);

*d* is the actual adsorbed water film thickness, calculated from d=0.012·RH−0.3 (based on adsorption isotherm experiment fitting, $R^{2}=0.96$).

The polarization effect of the liquid water microregion is reflected by increasing the thickness of adsorbed water film d and polarization attenuation coefficient λ. In the high humidity range (90~100% RH), the calculated value of d increases nonlinearly with the increase of RH, which indirectly reflects the dielectric contribution of liquid water micro region.

Combining Equation (7), the contribution of adsorbed water to the polarization strength *P*_4_ can be obtained as follows:

(10) $$P_{4}=N_{4}\alpha_{4}\varepsilon_{0}E_{loc}$$

Among them, *N_A_* is the number of dipoles per unit volume of adsorbed water, and *α_A_* is the polarizability of adsorbed water molecules. Similarly, *N_A_α_A_* is represented as a constant *M*:

(11) $$P_{4}=M\varepsilon_{0}E_{loc}$$

The total polarization intensity ***P****_total_* is the sum of the polarization intensities of each component:

(12) $$P_{toal}=P_{1}+P_{2}+P_{3}+P_{4}$$

Substituting Equations (5), (10), and (11) into the total polarization intensity Equation (11), combined with the volume fraction constraint ($v_{1}+v_{2}+v_{3}+v_{4}=1$), the final modified four phase mixed dielectric model is obtained:

(13) $$\ln\varepsilon_{r}=v_{1}\ln\varepsilon_{1}+v_{2}\ln\varepsilon_{2}+v_{3}\ln\left(1+k_{1}H\right)+v_{4}\ln\left(\varepsilon_{li}q_{ui}d/d_{0}\right)$$

Among them, $\varepsilon_{3}=1+k_{1}H$ (retaining the expression for the permittivity of raw water vapor, *k*_1_ is the original model parameter); the remaining parameters are consistent with the above definition.

## 4. Experimental Materials and Methods

### 4.1. Experimental Materials and Characterization

**Figure 2:**
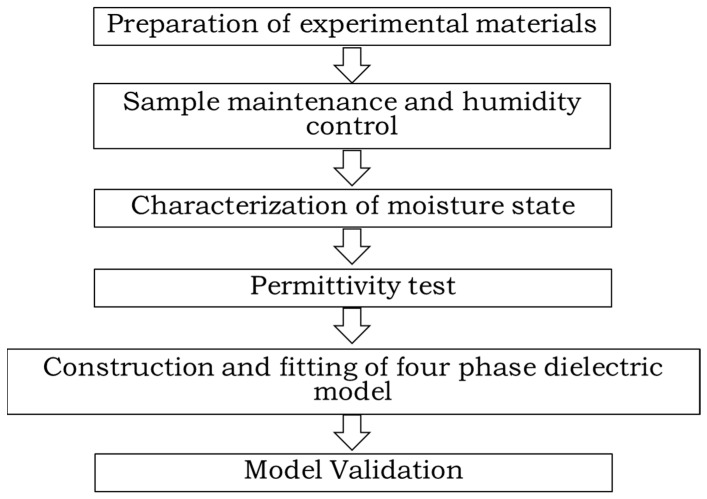
Roadmap for Experimental Research Technology.

In order to better carry out the experimental research, we have developed the experimental research technology roadmap, as shown in Figure 2.

The raw materials used to prepare ordinary cement concrete include cement, crushed stone, river sand, and water. The key characterizations of each material are as follows:

(a) Cement: in this study, ordinary Portland cement with strength grades of 32.5 and 42.5 were selected from Xinxiang Xinxing cement plant in Henan Province. The following performance tests are carried out on cement according to the relevant specifications. See Table 1 and Table 2 for the various performance indexes of cement.
(b) Aggregate: fine aggregate is selected from zone II medium sand with a fineness modulus of 2.7. The coarse aggregate is from Foguangdongshan quarry in Yanshi City, Henan Province. The particle size is divided into three gradations, namely 5–10 mm, 10–20 mm, and 16–31.5 mm. The aggregate gradation is determined by reference to the specifications. When the mixing ratio of coarse aggregate is 5–10:10–20:16–31.5 = 15:55:30, the maximum bulk density reaches 1703 kg/m^3^. See Table 3 for the material performance test results.
(c) Water: in this experiment, the mixed water is tap water in Zhengzhou city.

### 4.2. Specimen Preparation

(a) Cement mortar specimens: water cement ratio (*w*/*c*) 0.4, 0.5, 0.6, three parallel specimens in each group, size 100 × 100 × 100 mm, standard curing (temperature 20 ± 2 °C, relative humidity ≥ 95%) to 7 days, 28 days, 90 days;
(b) Pore structure of mortar/concrete: mix Proportion of Cement-based Composites is shown in Table 4; the pore structure of mortar/concrete at 28 days of age was measured by Mercury Intrusion Porosimetry (MIP), and the results are shown in Table 5, including total porosity, average pore diameter, and pore size distribution characteristics.
(c) Aggregate test piece: select a piece of limestone block with smooth and clean surface and dry it for 48 h.

### 4.3. Experimental Equipment and Test Methods

**Figure 3:**
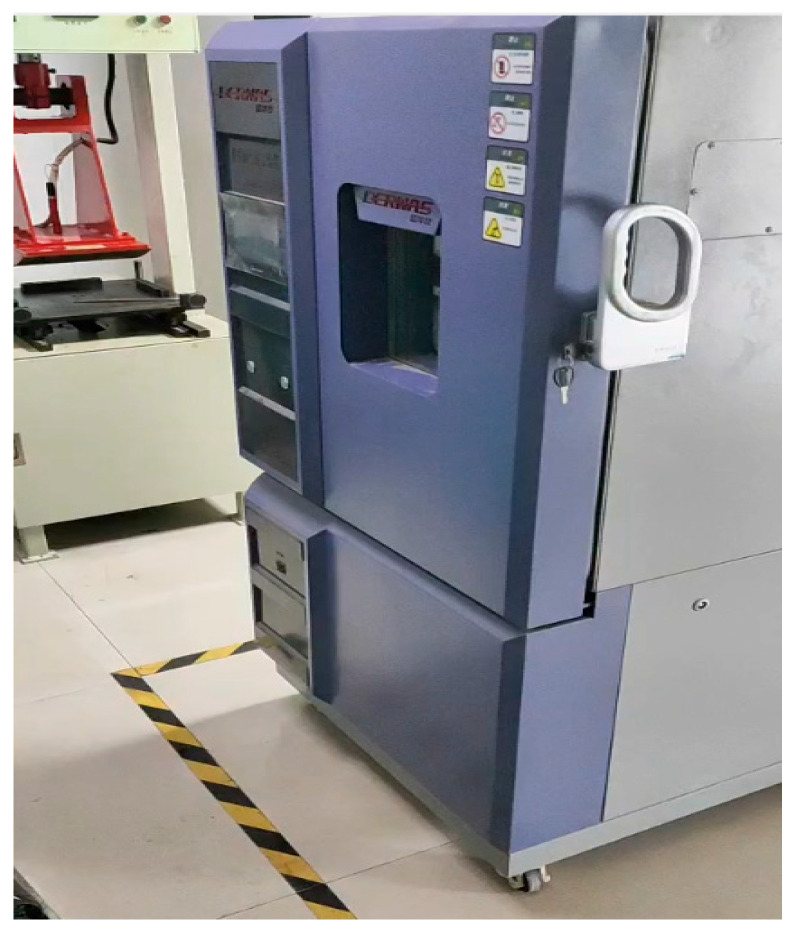
Constant temperature and humidity device.

**Figure 4:**
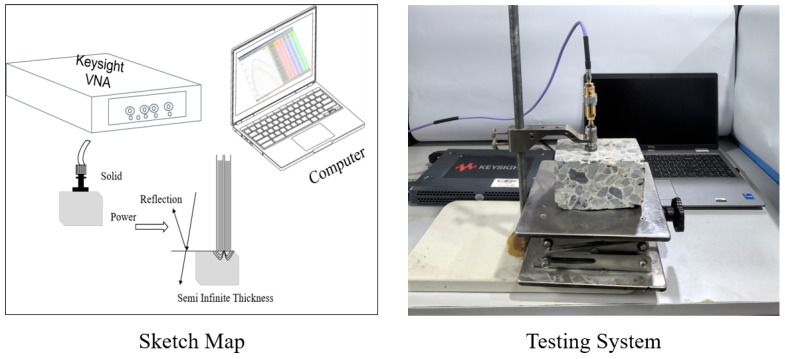
Coaxial probe method measurement system.

(a) Environmental equipment: During the entire experiment, the temperature of the constant temperature and humidity chamber was strictly controlled at 20 ± 0.5 °C, and real-time monitored by a high-precision temperature sensor (accuracy ±0.1 °C) built in the chamber, as shown in Figure 3. During the permittivity test, the surface temperature of the sample was ensured to be consistent with the chamber temperature (temperature difference ≤ 0.3 °C) to avoid the influence of temperature fluctuations on the test results.
(b) Permittivity test equipment: the Agilent P5001A network analyzer is preferred in this study. Its test frequency is 100 KHz~8 GHz, which can accurately measure the permittivity of various forms of substances. The equipment is from Shide Technology Co., Ltd., Santa Rosa, CA, USA. The permittivity of limestone aggregate, cement mortar, and cement concrete in dry state were measured by the coaxial probe method, and their permittivity under different humidity was measured. The test system is shown in Figure 4.
(c) Test frequency: 2 GHz (GPR common test frequency, considering penetration depth and accuracy)
(d) Humidity conditions: the relative humidity (RH) was set to 50%, 60%, 70%, 80%, 90%, and 100%. Based on the saturated water vapor pressure at 20 °C (2.339 kPa), the corresponding absolute humidity was recalculated as follows: 10.32 g/m^3^ (50% RH), 12.38 g/m^3^ (60% RH), 14.45 g/m^3^ (70% RH), 16.51 g/m^3^ (80% RH), 18.58 g/m^3^ (90% RH), and 20.64 g/m^3^ (100% RH). This humidity range fully covers the atmospheric humidity range in most areas of China and includes the high humidity saturation state of 90~100%, which is highly consistent with the humidity conditions of cement concrete pavements in actual engineering detection scenarios, such as rainy seasons and high-humidity environments, ensuring that the research results can be directly applied to the humidity correction of non-destructive ground-penetrating radar testing at engineering sites.
(e) Test steps: after the specimen is cured to the specified age, it is put into a constant temperature and humidity box and cured for 30 days under six kinds of humidity (to ensure the internal water and gas balance). The network analyzer is used to measure the permittivity, and the average value of three parallel specimens is taken as the test result.
(f) In this study, the test repeatability was strictly controlled. Three parallel samples were prepared for all cement-based composite samples, and each parallel sample was tested for dielectric constant for three times. During the test, the contact pressure between the probe and the sample surface is accurately controlled to 0.5 MPa by the constant pressure probe fixture to ensure that the contact state of each test is consistent and reduces the test error. The relative standard deviation (RSD) of all test data was calculated, and the validation results showed that the RSD of all test groups was ≤2.8% (lower than the 3.0% repeatability requirement of conventional test), indicating that the test data in this study had good repeatability and high reliability, which could be used as an effective basis for model construction and validation.

### 4.4. Moisture State Characterization

(a) Mass Change Monitoring: specimens cured to the specified age were placed in a constant temperature and humidity chamber and cured to mass stability (mass change rate ≤ 0.05% for 72 consecutive hours) under each RH condition. The mass change was recorded to calculate the moisture adsorption capacity (Δm = m_n_ − m_0_, where m_0_ is the mass in the dry state and m_n_ is the stable mass under each RH).
(b) Adsorption Isotherm Test: The adsorption isotherm of cement concrete at 20 °C was determined by the static gravimetric method, with an RH range of 50~100%. The BET model and Harkins–Jura model were fitted to determine the monolayer adsorbed water content and multi-layer adsorbed water content.

The values *v*_4_ (adsorbed water volume fraction) and film thickness *d* can be obtained by combining the data in Table 6. *d* is the average value of the effective adsorbed water film thickness in the 70~100% RH range at 20 °C. Since the variation trend of the d value in each RH range is consistent, the average value is used as the model fitting parameter to simplify the calculation and does not affect the prediction accuracy.

## 5. Results and Discussion

### 5.1. Variation Laws of Permittivity with Humidity

The permittivity of limestone aggregate, cement mortar, and cement concrete are measured by the coaxial probe method through the Agilent P5001A network analyzer. Before testing, the test frequency and calibration parameters were set in the software interface. Calibration with TRL: the permittivity of air, the short circuit breaker, and 20 °C purified water are tested with the coaxial probe in order to correct the possible directionality, tracking, and signal source mismatch errors in the reflection measurement. When measuring the permittivity of the dielectric surface, the probe is in close contact with the dielectric surface to measure its permittivity.

**Figure 5:**
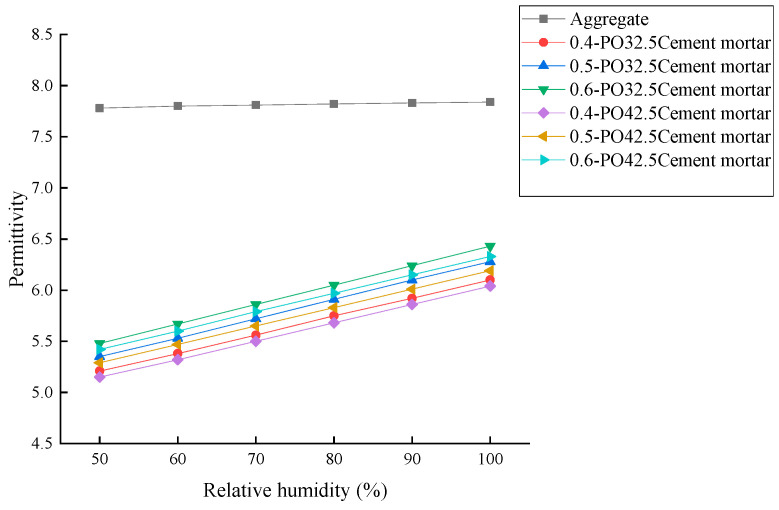
Permittivity of aggregates and cement mortar under different humidity conditions.

**Figure 6:**
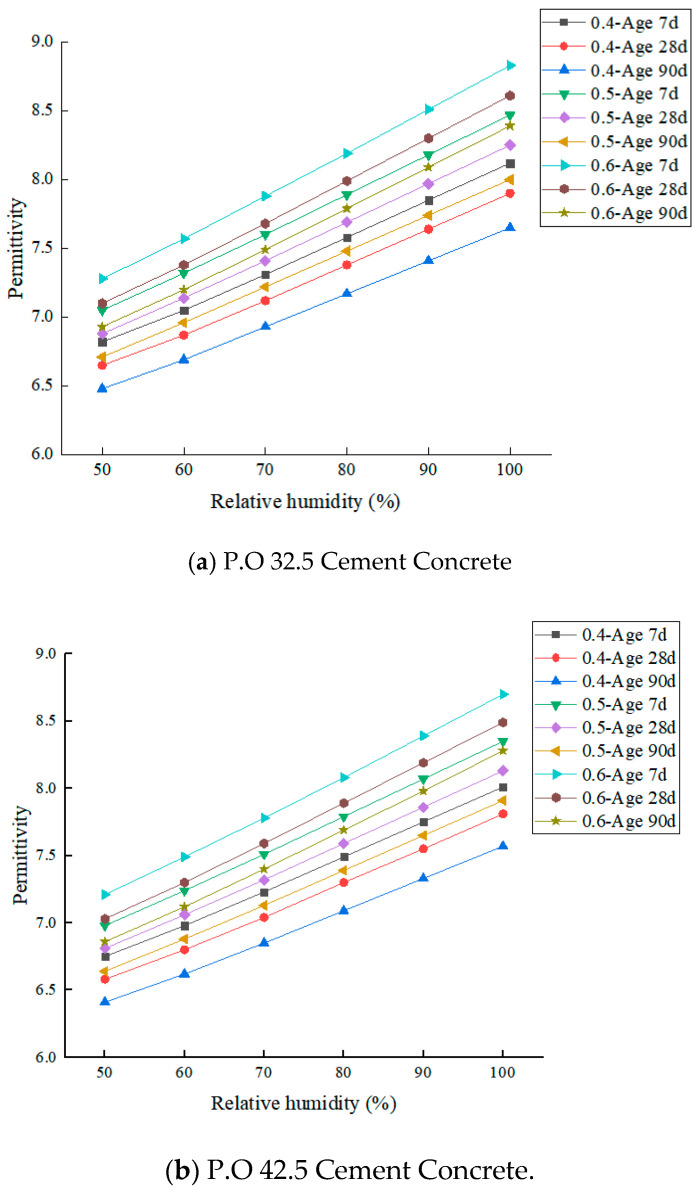
Permittivity of cement concrete at different ages under different humidity conditions.

According to the above test steps, the permittivity of limestone aggregate, cement mortar, and cement concrete under different absolute humidity conditions were measured, as shown in Figure 5 and Figure 6. The results are as follows:

(a) The permittivity of limestone aggregate is stable between 7.78 and 7.84 under different humidity conditions, and the maximum variation is only 0.77%. This phenomenon is consistent with the polarization mechanism of the aggregate, which is dominated by ionic polarization. The ionic polarization intensity is stable and less disturbed by environmental humidity, which verifies the intrinsic stability of the dielectric properties of the aggregate.
(b) The permittivity of cement mortar increases significantly and steadily with the increase of humidity. Taking P.O 32.5 cement mortar as an example, when *w*/*c* = 0.4, the relative humidity increased from 50% to 100%, and the permittivity increased from 5.21 to 6.10, an increase of 17.1%. When *w*/*c* = 0.6, the dielectric constant increased from 5.48 to 6.43, an increase of 17.3%, and the permittivity of cement mortar with a different water–cement ratio increased by the same range. This is because there is a large number of capillary pores in the cement mortar, and water vapor is easily adsorbed on the pore surface. The polarization process is the superposition of electron polarization and dipole polarization. The increase of humidity leads to the increase of water vapor concentration in the pores, and the dipole polarization contribution continues to increase, which finally shows the monotonic increase of permittivity.
(c) The results show that the permittivity of cement concrete with all mix proportions increases monotonously with the increase of relative humidity, and the growth rate exhibited a pattern of “slow at low humidity and accelerated at high humidity”. Taking P.O 32.5 cement concrete (*w*/*c* = 0.6, 28 *d* age) as an example, the dielectric constant increased from 7.10 to 7.68 in the range of 50% to 70% relative humidity, with an increase of 6.2%. The 70–100% range increased from 7.68 to 8.61, an increase of 14.4%, and the growth rate in the high humidity range was 2.2 times that of the low humidity range. This rule is consistent with the water vapor polarization mechanism: under low humidity, the water vapor in the pores exists in a dispersed state, and the dipole polarization contribution is limited. Under high humidity, pore water and gas reach the saturation state, and dipole polarization superposition forms a continuous polarization channel, which significantly improves the overall polarization intensity and leads to the rapid growth of permittivity.
(d) Under the same cement type, age, and humidity conditions, the permittivity of concrete increases with the increase of the water–cement ratio, and the humidity sensitivity increases simultaneously. Taking the 28 *d* age of P.O 42.5 cement concrete as an example, when the relative humidity is 100%, *w*/*c* = 0.4, The permittivity of 0.5 and 0.6 was 7.81, 8.13, and 8.49, respectively, which increased by 4.1% and 4.4%, respectively. This is because the larger the water–cement ratio, the higher the capillary porosity in the concrete, the stronger the water vapor adsorption capacity and storage capacity, and the more significant the response of dipole polarization to the change of humidity, which ultimately shows the increase of dielectric permittivity.
(e) With the increase in age, the permittivity of concrete decreases slightly. Taking the concrete with P.O 32.5 *w*/*c* = 0.5 as an example, the permittivity of 7-day-old concrete with 100% relative humidity is 8.47, which decreases to 8.25 at 28 days, further decreases to 8.00 at 90 days, and decreases by 5.5% at 90 days compared with that at 7 days. This phenomenon stems from the fact that the increase in age promotes the continuous hydration of cement, and the hydration products constantly fill the internal capillary pores, reducing the effective sites of moisture adsorption, reducing the effect of humidity on the polarization intensity, and making the dielectric properties of concrete more stable.

### 5.2. Model Fitting and Independent Validation

#### 5.2.1. Model Fitting

Specimens were prepared according to the mix proportion in Table 7, and the test data of four points (50%, 60%, 80%, 100% RH) were selected for model fitting to determine the model parameters. The fitting results are shown in Table 6, with R^2^ > 0.95.

**Figure 7:**
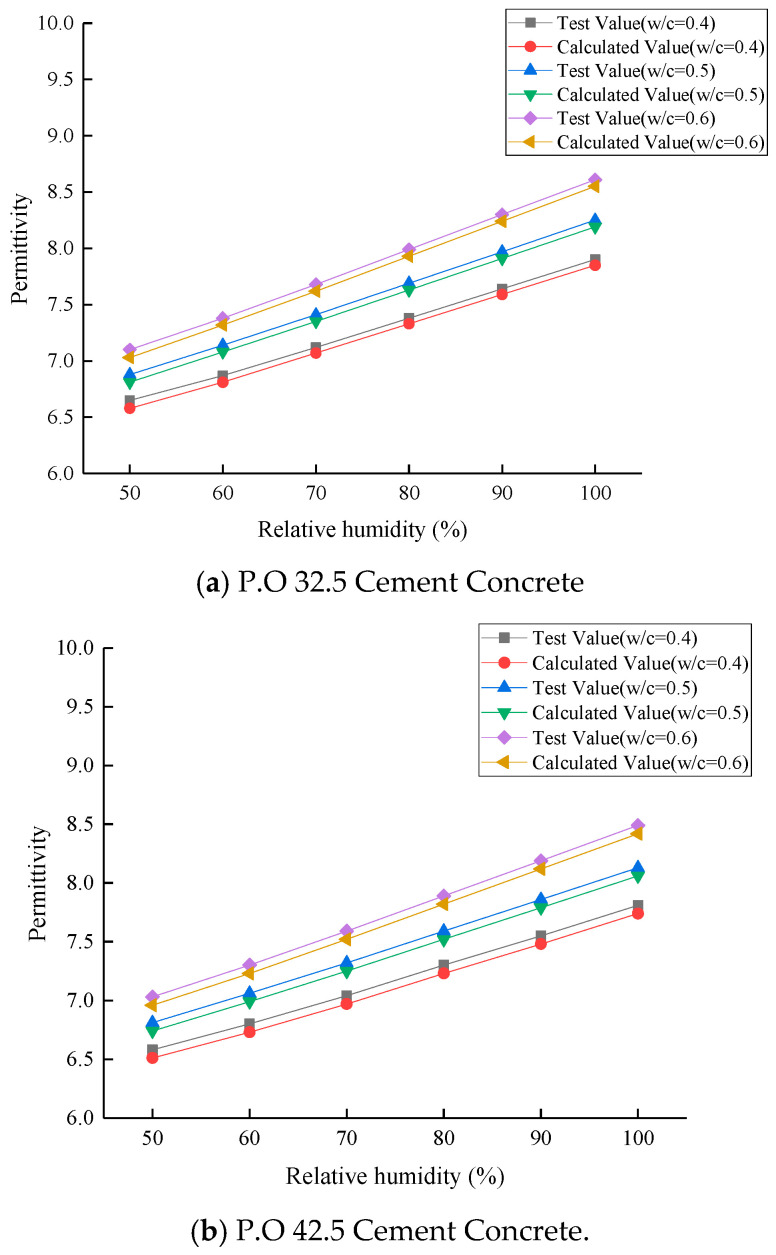
Fitting curve between permittivity and absolute humidity of 28 d old cement concrete.

**Figure 8:**
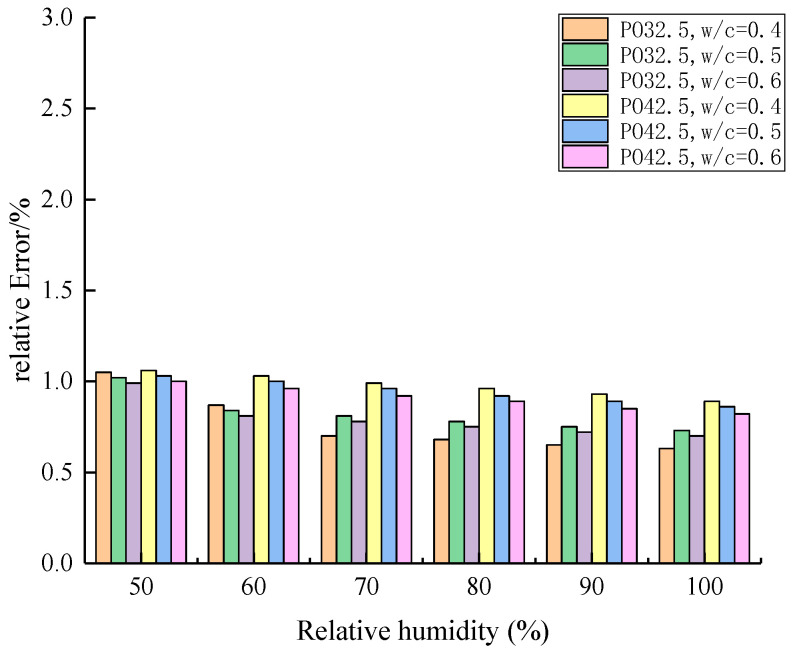
Comparison of calculated and measured permittivity of 28 d old cement concrete.

At the age of 28 days, the comparison between the calculated and measured values of the permittivity model of cement concrete with different cement types and water–cement ratio is shown in Figure 7 and Figure 8. The relative errors are less than 5%, which verifies the reliability of the model.

#### 5.2.2. Independent Validation

(a) Hold-out Validation: two points (70% and 90% RH) not involved in fitting were used as the validation set. The permittivity was predicted and compared with the measured values, as shown in Table 8, with relative error < 4%. (b) Independent Batch Validation: a new batch of specimens with the same mix proportion (independent mixing and curing) was prepared, and the permittivity was tested over the entire RH range as independent validation data, with a predicted relative error < 5%. (c) Prediction Interval: based on the normal distribution assumption of the model fitting residuals, the prediction interval ($PI=\overset{\wedge }{y}\pm t_{\left(n-p\right)}S_{e}\sqrt{1+1/n}$) at the 95% confidence level was calculated, where *ŷ* is the predicted value, *t*_(*n*−*p*)_ is the t-distribution critical value, *S_e_* is the standard error, n is the number of samples, and *p* is the number of model parameters. The prediction interval results are shown in Table 8, and all measured values fall within the 95% prediction interval. Based on the normality test results of the model fitting residuals, the prediction interval at the 95% confidence level was calculated, and all measured values fell within this interval, indicating that the prediction results of the model are reliable.

### 5.3. Influence of Different Factors on Humidity Sensitivity

The age of cement concrete will change the moisture diffusion path and accumulation state by affecting the degree of hydration and pore structure (porosity, pore size distribution), which will lead to differences in humidity sensitivity. In this section, three groups of cement concrete specimens with the same mix ratio (water–cement ratio 0.5) of 7 d, 28 d, and 90 d are selected to compare the humidity sensitivity laws of different ages through tests and model analysis.

#### 5.3.1. Humidity Sensitivity Evaluation Index

The humidity sensitivity coefficient is used to evaluate the humidity sensitivity of cement concrete, which is defined as when the absolute humidity increases from 10.32 g/m^3^ to 20.64 g/m^3^. Equation (14) for calculating the permittivity growth rate can be obtained as follows:

(14) $$S_{H}=\frac{\varepsilon_{\max}-\varepsilon_{\min}}{\varepsilon_{\min}}\times 100\%$$

where $\varepsilon_{\max}$ is the permittivity at an absolute humidity of 30.31 g/m^3^; $\varepsilon_{\min}$ is the permittivity at absolute humidity of 15.16 g/m^3^.

#### 5.3.2. Humidity Sensitivity Comparison Results

Calculate the humidity sensitivity coefficient of cement concrete at different ages according to Equation (14), as shown in Table 9.

According to the results in Table 4, the following conclusions can be drawn:

(a) The humidity sensitivity coefficient of all experimental groups decreased with age; the maximum value was at 7 days, and the minimum was at 90 days. The reason is that the longer the age, the more sufficient the hydration of the cement, and the hydration products fill the pores, making the material structure denser, increasing the water vapor diffusion resistance, and weakening the influence of humidity on the dielectric properties.
(b) Under the same cement type and age, the greater the water cement ratio, the greater the humidity sensitivity coefficient. This is because the larger the water–cement ratio, the higher the porosity, the stronger the water vapor adsorption and diffusion ability, and the dielectric properties are more sensitive to humidity changes.
(c) Under the same water–cement ratio and age, the humidity sensitivity coefficient of P.O 32.5 cement concrete is slightly higher than P.O 42.5. The reason is that the hydration reaction of P.O 42.5 cement is more sufficient, the product structure is more compact, the porosity is lower, and the humidity sensitivity is relatively weak.

## 6. Conclusions

Through systematic experiments and theoretical analysis, this study reveals the influence mechanism of relative humidity on the dielectric properties of CBC, constructs a dielectric model considering multiple moisture forms, and verifies its application value in the humidity correction of GPR. Based on the results of this study, the following conclusions can be drawn:

(a) It is clear that the core mechanism of humidity affecting the dielectric properties of CBC is the synergistic effect of dipole polarization of water vapor and multilayer polarization of adsorbed water: in the low humidity range, the weak polarization of dispersed water vapor and monolayer adsorbed water is dominant, and the permittivity increases slowly; in the middle and high humidity range, multilayer adsorbed water forms a continuous water film, and polarization superposition accelerates the growth of permittivity; and capillary condensation in macropores in high humidity region forms liquid water microregion, which significantly enhances the polarization intensity and rapidly increases the permittivity.
(b) Based on the modification of the original three-phase model, a four-phase dielectric model of “cement mortar aggregate steam adsorbed water” was constructed. The model inherited the framework of the classic Brown hybrid model and added the adsorbed water phase parameters. The goodness of fit of the model R^2^ > 0.94, and the relative error between the calculated value and the measured value is less than 5%. The dielectric model established in this study shows good prediction accuracy and stability under the condition of representative mixture ratio. It should be noted that the current verification scope is mainly based on representative specimens under laboratory-controlled conditions, and the applicability of the model under different cement grades, water–cement ratio variation range, and long-term service age still needs to be systematically verified through a wider range of experimental data.
(c) Moisture sensitivity of cement-based composites is controlled by the water–cement ratio and cement strength grade and age. The higher the water–cement ratio and the lower the cement strength grade, the stronger the humidity sensitivity. The sensitivity was decreased by 5~6% at 90 days compared with that at 7 days.
(d) The four-phase dielectric model constructed in this study complements the humidity correction framework proposed by Zhong et al. [25] and provides a more comprehensive theoretical support for GPR field detection. The polarization synergy effect of water morphology in the model is also mutually confirmed with the long-term monitoring results of Shen et al. [16] on the dielectric properties of cement-based materials.
(e) The GPR humidity correction scheme based on this model will provide a reliable tool for the humidity interference correction of GPR detection in engineering practice, and will help to solve the problem of low accuracy of GPR quantitative interpretation of cement concrete structures for a long time.

## Figures and Tables

**Table 1:** Physical properties of cement.

Cement Grade	Project	Apparent Density (g/cm^3^)	Specific Surface Area (kg/m^3^)	Setting Time/min		Compressive Strength/MPa		Flexural Strength/MPa	
				Initial Setting Time	Final Setting Time	3 d	28 d	3 d	28 d
P.O 32.5	Test value	2.89	328.4	140	169	28.9	37.8	4.6	6.3
	Standard value	2.85–3.05	≥300	≥45	≤600	≥12	≥32.5	≥3	≥5.5
P.O 42.5	Test value	3.03	336.1	146	175	36.1	50.1	6.9	8.7
	Standard value	2.8–3.1	≥300	≥45	≤600	≥17	≥42.5	≥3.5	≥6.5

**Table 2:** Chemical composition of cement (%).

Cement Grade	CaO	SiO_2_	Al_2_O_3_	SO_3_	Fe_2_O_3_	MgO	Na_2_O	LOI
P.O 32.5	62.852	21.537	6.345	2.578	3.247	2.1	0.1	1.635
P.O 42.5	67.740	17.444	5.796	3.695	3.521	2.868	0.2	1.744

**Table 3:** Physical properties of aggregates.

Aggregate	Fineness Modulus	Bulk Density (kg/m^3^)	Apparent Density (kg/m^3^)	Crushing Value (%)	Moisture Content (%)	Water Absorption (%)
Coarse aggregate	—	1486	2780	8.7	0.1	0.7
Fine aggregate	2.7	1596	2571	—	3.1	1.3

**Table 4:** Mix Proportion of Cement-based Composites (Unit: kg/m^3^).

Cement Type	Water–Cement Ratio	Cement	Water	Coarse Aggregate (5~20 mm)	Fine Aggregate (Medium Sand)	Volume Fraction of Cement Mortar (%)	Volume Fraction of Aggregate (%)	Initial Porosity (%)	Aggregate Moisture Content (%)	Actual Mixing Water (kg/m^3^)
P.O 32.5	0.4	450	180	855	510	68.2	30.0	1.8	0.5	163.3
	0.5	420	210	860	505	69.5	30.0	0.5	0.5	193.5
	0.6	390	234	865	500	70.8	30.0	0.2	0.5	217.6
P.O 42.5	0.4	460	184	850	515	67.9	30.0	2.1	0.5	167.2
	0.5	430	215	855	510	69.2	30.0	0.8	0.5	198.3
	0.6	400	240	860	505	70.5	30.0	0.5	0.5	223.5

**Table 5:** Pore Structure Characterization Results of Mortar/Concrete at 28 Days of Age.

Sample Type	Water–Cement Ratio	Total Porosity (%)	Average Pore Diameter (nm)	Proportion of Pores < 100 nm (%)
P.O 32.5 Mortar	0.4	18.2	85	68.5
	0.5	21.5	102	62.3
	0.6	25.8	128	55.7
P.O 42.5 Mortar	0.4	17.5	80	70.2
	0.5	20.8	95	64.5
	0.6	24.5	120	58.3
P.O 32.5 Concrete	0.4	14.8	90	75.3
	0.5	15.6	98	72.1
	0.6	16.8	105	69.8
P.O 42.5 Concrete	0.4	13.5	85	78.5
	0.5	14.3	82	75.4
	0.6	15.5	92	72.6

**Table 6:** Water absorption of cement-based composites (28 d, 20 °C).

Cement Type	Water–Cement Ratio	Single Layer Water Absorption (BET) (mg·g^−1^)	Single Layer Water Absorption SD (mg·g^−1^)	Multilayer Water Absorption (Harkins–Jura Fitting) (mg·g^−1^)	Multilayer Water Absorption SD (mg·g^−1^)	Total Water Absorption (mg·g^−1^)	BET Fitting (R^2^)	Harkins–Jura Fitting (R^2^)
P.O 32.5	0.4	2.21	0.05	5.84	0.12	8.05	0.989	0.982
	0.5	2.65	0.06	7.52	0.15	10.17	0.987	0.979
	0.6	3.28	0.07	8.51	0.17	11.82	0.985	0.978
P.O 42.5	0.4	2.15	0.05	5.62	0.11	7.77	0.990	0.983
	0.5	2.58	0.06	7.15	0.14	9.73	0.988	0.981
	0.6	3.12	0.07	8.20	0.16	11.32	0.0986	0.980

**Table 7:** Fitting Parameters of Dielectric Model for 28 *d* Age Cement Concrete.

Sample Type	Water–Cement Ratio	*v*_1_ (%)	*v*_2_ (%)	*v*_3_ (%)	*v*_4_ (%)	*d* (nm)	*k*	R^2^
P.O 32.5	0.4	68.2	30.0	1.0	0.85	1.0	0.92	0.986
	0.5	69.5	30.0	0.7	0.92	1.0	0.95	0.989
	0.6	70.8	30.0	0.4	1.05	1.0	0.98	0.983
P.O 42.5	0.4	67.9	30.0	1.1	0.78	1.0	0.90	0.984
	0.5	69.2	30.0	0.8	0.86	1.0	0.93	0.987
	0.6	70.5	30.0	0.5	0.98	1.0	0.96	0.981

**Table 8:** Independent validation results of the model (P.O 32.5 concrete, *w*/*c* = 0.5, 28 days).

RH (%)	Measured ε’	Fitted Predicted ε’	Independent Predicted ε’	Relative Error (%)	95% Prediction Interval
70 (Validation Set)	7.45	-	7.38	0.94	[7.21, 7.55]
90 (Validation Set)	8.12	-	8.085	0.86	[7.88, 8.22]
50 (Independent Batch)	7.10	-	7.05	0.71	[6.88, 7.22]
80 (Independent Batch)	7.83	-	7.79	0.51	[7.62, 7.96]

**Table 9:** Humidity sensitivity coefficient of cement concrete at different ages $S_{H}$ (%).

Cement Model	Water–Cement Ratio	7 d	28 d	90 d	Sensitivity Trend
P.O 32.5	0.4	30.7	28.9	27.1	7 d > 28 d > 90 d
	0.5	33.7	32.0	29.8	7 d > 28 d > 90 d
	0.6	35.8	34.5	32.3	7 d > 28 d > 90 d
P.O 42.5	0.4	29.8	27.5	25.6	7 d > 28 d > 90 d
	0.5	32.6	30.4	28.2	7 d > 28 d > 90 d
	0.6	34.7	32.8	30.5	7 d > 28 d > 90 d

## Data Availability

The original contributions presented in this study are included in the article. Further inquiries can be directed to the corresponding author.
